# Regulating the Regulators: The Role of Histone Deacetylase 1 (HDAC1) in Erythropoiesis

**DOI:** 10.3390/ijms21228460

**Published:** 2020-11-11

**Authors:** Min Young Kim, Bowen Yan, Suming Huang, Yi Qiu

**Affiliations:** 1 Department of Cellular and Molecular Physiology, Pennsylvania State University College of Medicine, Hershey, PA 17033, USA; mkim6@pennstatehealth.psu.edu (M.Y.K.); byan@pennstatehealth.psu.edu (B.Y.); 2 Department of Pediatric, Pennsylvania State University College of Medicine, Hershey, PA 17033, USA; shuang4@pennstatehealth.psu.edu; 3 Penn State Cancer Institute, Pennsylvania State University College of Medicine, Hershey, PA 17033, USA

**Keywords:** erythropoiesis, histone deacetylase, HDAC1, GATA-1, PU.1, hemoglobin, reactivation of fetal globin, HDAC inhibitor

## Abstract

Histone deacetylases (HDACs) play important roles in transcriptional regulation in eukaryotic cells. Class I deacetylase HDAC1/2 often associates with repressor complexes, such as Sin3 (Switch Independent 3), NuRD (Nucleosome remodeling and deacetylase) and CoREST (Corepressor of RE1 silencing transcription factor) complexes. It has been shown that HDAC1 interacts with and modulates all essential transcription factors for erythropoiesis. During erythropoiesis, histone deacetylase activity is dramatically reduced. Consistently, inhibition of HDAC activity promotes erythroid differentiation. The reduction of HDAC activity not only results in the activation of transcription activators such as GATA-1 (GATA-binding factor 1), TAL1 (TAL BHLH Transcription Factor 1) and KLF1 (Krüpple-like factor 1), but also represses transcription repressors such as PU.1 (Putative oncogene Spi-1). The reduction of histone deacetylase activity is mainly through HDAC1 acetylation that attenuates HDAC1 activity and trans-repress HDAC2 activity through dimerization with HDAC1. Therefore, the acetylation of HDAC1 can convert the corepressor complex to an activator complex for gene activation. HDAC1 also can deacetylate non-histone proteins that play a role on erythropoiesis, therefore adds another layer of gene regulation through HDAC1. Clinically, it has been shown HDACi can reactivate fetal globin in adult erythroid cells. This review will cover the up to date research on the role of HDAC1 in modulating key transcription factors for erythropoiesis and its clinical relevance.

## 1. Introduction

Histone deacetylases (HDACs) catalyze the removal of acetyl groups from the ε-amino groups of lysine residues. The reversible acetylation of histones and non-histone proteins by histone acetyltransferases (HATs) and HDACs play critical roles in transcriptional regulation in eukaryotic cells. Histone acetylation is commonly associated with transcriptional activation of genes, and is thought to be responsible for the formation of a local “open chromatin” structure required for the binding of multiple transcription factors (Reviewed in [1]). In contrast, the removal of acetyl groups by histone deacetylases frequently accompanies the suppression of gene activity (Reviewed in [2]). However, non-histone protein lysine acetylation plays a diverse role in the regulation of all aspects of cellular processes that may result in transcription activation or repression [3,4,5,6].

Mammalian HDACs are classified into four classes (I, II, III, and IV) based on the sequence homology of the yeast histone deacetylases Rpd3 (reduced potassium dependency), Hda1 (histone deacetylase 1), and Sir2 (silent information regulator 2), respectively. Class I HDACs include HDAC1, 2, 3 and 8, class II HDACs contain HDACs 4, 5, 6, 7, 9 and 10. Class III enzymes, however, require the coenzyme NAD+ (Nicotinamide adenine dinucleotide) as a cofactor. HDAC11 belongs to the class IV family. Class I HDACs are ubiquitously expressed nuclear proteins. Although these enzymes share a high level of sequence homology and common substrates, and HDAC2 can partially compensate for HDAC1 loss, each of the enzymes has a unique role in cell function, as deletion of each member of the class I deacetylase leads to lethality (reviewed in [7]). HDAC1 knockout mice die before embryonic day 10.5 with severe growth defects [8,9]. The total deacetylase enzymatic activity is greatly reduced in HDAC1 null ES cells, indicating that HDAC1 is a major deacetylase in ES cells [10]. Conditional HDAC1/2 double knockout mice show significant hematopoietic defect, mainly in the erythroid/megakaryocyte compartment [11]. Hematopoietic deficiency has also been reported in clinical trials with various HDACi (HDAC inhibitors), including anemia, thrombocytopenia, neutropenia, and lymphopenia (reviewed in [12]), indicating that HDACi affects hematopoiesis in multiple lineages. Therefore, it is extremely important to understand how HDAC affects normal hematopoiesis.

## 2. HDAC1: Modifications and Associated Corepressor Complexes

### 2.1. Histone Deacetylase 1 (HDAC1) and Its Associated Complexes 

HDAC1 is found in at least three evolutionally conserved, distinct protein complexes: the Sin3, the CoREST and the Mi-2/NuRD complexes (Reviewed in [2,13]). HDAC1 and HDAC2 are highly related enzymes with 82% overall sequence identity and often coexist in these complexes [14,15,16]. All complexes are recruited to target genes through interactions with DNA binding transcription factors. Sin3, originally identified as the transcription corepressor of Mad-Max (Max dimerization protein, Myc-associated factor X), requires HDAC1 and HDAC2 deacetylase activities for its full repression [17,18,19,20]. Sin3 complexes also contain RbAp46/48 (retinoblastoma associated protein-46/48) [18], SAP18 (Sin3A Associated Protein 18) and SAP30 (Sin3A Associated Protein 30) [21]. The Sin3 complex may also contain ING1 (Inhibitor of Growth Family Member 1), RBP1 (Retinoblastoma-binding protein 1), RBP-like protein SAP180 (Sin3A Associated Protein 180), SDS3 (Suppressor of defective silencing 3) and BRMS1 (Breast cancer metastasis suppressor 1) [22,23,24,25,26]. Sin3 has been identified in most eukaryotes; it contains the conserved basic structure of multiple PAH (Paired amphipathic helix) domains for protein-protein interaction. Sin3 does not bind to DNA and has no known enzymatic activity of its own. It may function, however, through its ability to interact with other proteins [27]. Mammals have two isoforms, Sin3A (SIN3 Transcription Regulator Family Member A) and Sin3B (SIN3 Transcription Regulator Family Member B), which provide more diverse protein complexes for gene regulation. 

The CoREST complex is a multi-subunit complex containing the lysine demethylase LSD1 (Lysine-specific demethylase 1), corepressor CtBP (C-terminal binding protein), BHC80 (BRAF35-HDAC complex protein BHC80), CoREST, HDAC1 and HDAC2 [28,29,30,31,32]. LSD1 can demethylate mono- or di-methyl histone H3 lysine 4 or lysine 9 for gene repression or activation [33,34,35]. Thus, this complex is capable of deacetylating as well as demethylating nucleosomes. CoREST is required to tether HDAC1 to nucleosomes for nucleosomal deacetylation [35]. Interestingly, LSD1 can further enhance deacetylation on nucleosomes, suggesting that LSD1 demethylase activity is required for optimal deacetylase activity. In reverse, deacetylation of nucleosomes is required for LSD1 enzymatic activity [29], thus unveiling the functional cross-talk between the demethylase and deacetylase. 

The NuRD complex includes the ATPase/helicase Mi-2, HDAC1/2, MTA (Metastasis-associated) proteins, MBD2 (methyl CpG-binding domain-2), and RbAp46/48 [36,37]. Mi-2 belongs to the CHD (chromo-helicase DNA-binding) protein family [38]. There are two isoforms of Mi-2, the Mi-2α(CHD3) and Mi-2β(CHD4), both of which are integral components of the NuRD complex. Further studies have shown that the NuRD complex is itself part of a larger protein complex of 10 subunits, the so-called MeCP1 (methyl CpG-binding protein 1) complex [39,40,41,42]. NuRD/MeCP1 is recruited to target genes through interactions with DNA binding transcription factors. Interestingly, a recent report suggests that LSD1 is also associated with the NuRD complex [43,44,45]. NuRD/MeCP1 is a repressor complex in most case. For example, the NuRD/MeCP1 complex is associated with gene repression during development [46]. However, interaction of GATA-3 with NuRD in differentiating Th2 (Type 2 helper T) cells was shown to activate transcription of the IL-4 (Interleukin 4) gene after displacing MBD2 from the chromatin [47]. NuRD is also required for GATA-1 mediated gene activation [48,49]. These observations suggest that Mi-2 containing NuRD or MeCP1 complexes can either repress or activate gene transcription.

In addition to these three complexes, HDAC1 also associates with other sub-complexes during development [13], including a variety of proteins, such as transcription factors, coactivators, chromatin remodeling proteins, etc., thus adding to the complexity of HDAC1 functions [13,50].

### 2.2. Regulation of HDAC1 Activity through Posttranslational Modification

HDAC1 activity is modulated through various post translational modifications including phosphorylation, acetylation, ubiquitination, sumoylation, and nitrosylation [51]. HDAC1 is phosphorylated at serine 421 and 423 by casein kinase II and dephosphorylated by mitogen-activated protein kinase phosphatase-3 (MKP-3) [52,53]. Mutagenesis analysis suggested that phosphorylation on these sites are important for histone deacetylase activity and for HDAC1 to interact with corepressor complexes [53]. However, treatment with phosphatases did not significantly affect HDAC1 histone deacetylase activity in vivo [52,53,54,55]. Therefore, it is suggested that phosphorylation may be required for initial folding and may be dispensable for subsequent functional events [56]. HDAC1 phosphorylation increases during the G1 phase and was significantly reduced during the late S/G2 phase [57]. It is unclear whether the increase of phosphorylation is linked to the increase of HDAC1 protein levels during the cell cycle [56]. There are also other minor sites that may be phosphorylated [55,58]. It remains to be elucidated whether phosphorylation on these sites affect HDAC1 function. 

**Figure 1:**
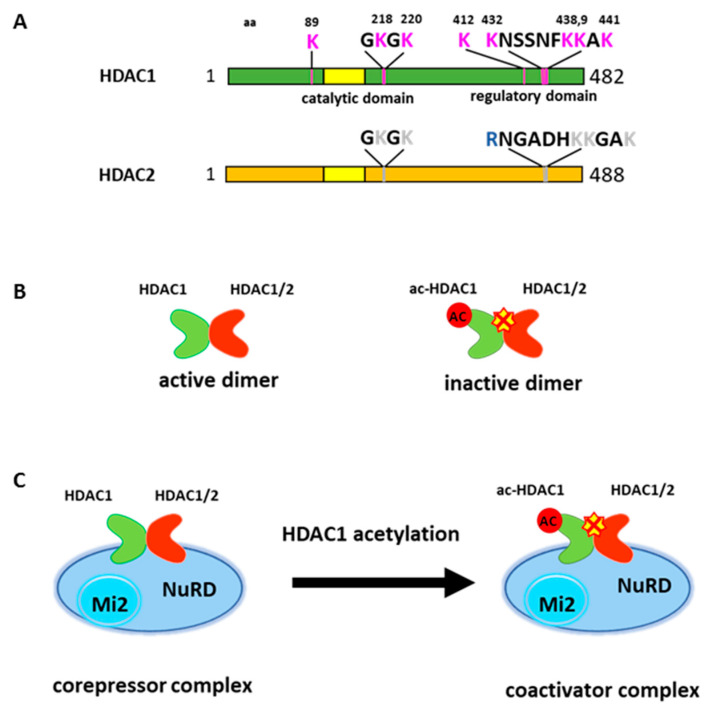
HDAC1 acetylation abolishes deacetylase activity and convers its associated corepressor complex to coactivator complex. (**A**) Schematic representation of HDAC1 acetylation sites. HDAC2 is not acetylated. Note lysine 432 in HDAC1 corresponds to an arginine in HDAC2. (**B**) HDAC1 and 2 exist as a homo or heterodimer. Acetylation on HDAC1 represses its deacetylase activity and results in inactive deacetylase dimer. (**C**) HDAC1 acetylation converse NuRD complex to a coactivator complex.

HDAC1 can also undergo acetylation modification. Qiu et al. (2006) initially reported HDAC1 acetylation at six lysine residues [6]. Two acetylated lysines (K218, 220) are located near the catalytic core domain and other four lysines (K432, 438, 439 and 441) are located at the C-terminal region of the protein (Figure 1A). The C-terminal domain does not have any catalytic activity. However, the deletion of this region greatly reduces deacetylase activity, therefore this region is considered a regulatory domain [18]. Acetylation on HDAC1 abolishes deacetylase activity in vitro and in vivo [6,59]. Lysine 432 acetylation seems to be crucial for subsequent acetylation on other lysines and appears to be important in regulating histone deacetylase activity [16,60]. The mutation on lysine 432 to glutamine, which mimics acetylated lysine, profoundly reduced histone deacetylase activity [6,16]. Subsequent studies found additional sites for acetylation, but the function of these acetylation sites remain elusive [60]. 

Unlike constitutive phosphorylation, acetylation on HDAC1 is an inducible event. Glucocorticoid receptor (GR) associated HDAC1 becomes acetylated by p300 when GR mediated transcription activation is down regulated on the GR responsive promoter [6]. Cellular stress, such as heat shock or DNA damage, can also induce HDAC1 acetylation to facilitate cellular response [60,61]. HDAC1 acetylation is also increased during erythroid differentiation to promote this process [49]. 

HDAC1 and HDAC2 often coexist in multi-component protein complexes and are highly related enzymes [14,15,16]. Interestingly, the major acetylation modifications of HDAC1 occur in the C-terminal domain, which reveals a lesser degree of homology to HDAC2. Further, the activity of HDAC2 is trans-repressed when forming heterodimer with acetylated HDAC1 [16] (Figure 1B). Thus, HDAC1 acetylation results in the inhibition of overall deacetylase activity within HDAC1 containing complexes (Figure 1C). 

## 3. HDAC1 Is a Master Regulator for Erythropoiesis

Blood cells consist of cells from multiple lineages, which are specialized for oxygen delivery, homeostasis, and defense from infection. Diverse lineages of blood cells originate from hematopoietic stem cells (HSC), which reside within the bone marrow. HSC give rise to multipotent progenitors (MPPs) that continue to differentiate into common myeloid progenitors (CMPs) and common lymphocyte progenitors (CLPs), which further differentiate toward the specific cell lineages. CMPs differentiate to generate megakaryocytes, red blood cells, neutrophils and macrophages, while CLPs differentiate into B cells and T cells [62,63]. 

Hematopoietic deficiencies such as anemia, thrombocytopenia, neutropenia, and lymphopenia have been reported in clinical trials with various HDACi [12], indicating the importance of HDACs in hematopoietic development. The role of HDAC1 in development was first studied in knockout mice. Deletion of *Hdac1* in mice results in embryonic lethality before E10.5 due to severe proliferation defects and retardation in development [9]. The *Hdac1/2* double knockout in hematopoietic compartment was established in mice harboring the interferon-inducible MxCre (Mx dynamin-like GTPase/Cyclic recombinase) transgene and conditional knockout alleles for *Hdac1* and/or *Hdac2*. The *Hdac1/2* double knockout mice die approximately nine days after pI/pC (Polyinosinic/polycytidylic acid) injections, displaying significant hematopoietic defects that reduced bone-marrow cell numbers and affected differentiation of all major hematopoietic lineages. The reduction of bone marrow cell numbers was associated with an increase in apoptotic bone marrow cells. The most significant defect was in erythroid/megakaryocyte compartment, including reduction of mature differentiated cells and increased apoptosis [11,64]. Individual knockout of *Hdac1* and *Hdac2* show compensatory and overlapping functions in hematopoiesis. However, *Hdac1* knockout mice expressing mono-allelic *Hdac2* showed severe erythropoiesis defect, with mice dying 12 days after pI/pC induction. The defect included reduction of proerythroblast and mature erythroblast in bone marrow and compensatory extramedullary hematopoiesis. *Hdac2* knockout mice expressing mono-allelic *Hdac1* developed normally, indicating the essential role of HDAC1 in erythropoiesis [64].

Experiments conducted in hematopoietic progenitor cells show that HDAC1 deacetylase activity plays diverse roles in erythropoiesis, including promoting cell survival and proliferation [65,66,67]. Importantly, HDAC1 associated deacetylase activity is significantly decreased during the process of erythropoiesis [68]. Consistently, HDAC1 acetylation levels increased after the induction of erythroid differentiation in multiple cell models, including in human CD36+ cells, mouse G1E erythroblast cells, and mouse erythroleukemia cells [49]. The acetylation of HDAC1 correlates to the reduction of overall histone deacetylase activity in the cell after the induction of differentiation. 

Erythropoiesis is regulated through lineage specific transcription factors for the proliferation and differentiation of multipotent progenitors into the erythroid lineage. Among them, GATA-1, GATA-2 (GATA-binding factor 2), FOG-1 (Friend of GATA Protein 1), TAL1/SCL/LMO2/Ldb1/E2A (TAL BHLH Transcription Factor 1/Stem cell leukemia/ LIM Domain Only 2/ LIM-domain-binding protein 1/ Early 2A protein), KLF1, Gfi-1b (Growth factor independent protein 1b), BCL11A (B-cell lymphoma/leukemia 11A), NF-E2 (Nuclear factor, erythroid-derived 2), and PU.1 are hematopoietic-specific transcription factors required for erythropoiesis [69]. Importantly, HDAC1 interacts with all factors and regulates their recruitment and transcription activity. Further, acetylated HDAC1 incorporates into their associated complex and regulates transcription activity, thus confirming HDAC1 as a master regulator for erythropoiesis. 

## 4. GATA-1 Interacts with HDAC1 Containing NuRD Complex for Activation and Repression

Hematopoietic lineage specific transcription factor GATA-1 is the founding member of the GATA factor family. GATA-1 is primarily expressed in erythroid lineage. It is also expressed in megakaryocytes, eosinophils, mast cells, and sertoli cells of the testis (reviewed in [70,71]). GATA-1 is essential for normal hematopoiesis, especially for erythropoiesis and megakaryopoiesis [72]. Mutations of the GATA-1 gene cause characteristic hematologic diseases [71,73]. GATA-1 knockout mice die between embryonic days 10.5 and 11.5 due to severe anemia. Embryonic stem cells or erythroid cells of mice deficient in GATA-1 fail to develop into mature erythroid cells [74,75,76]. GATA binding sites are present in the regulatory regions of virtually all erythroid-specific genes, suggesting that GATA-1 represents an erythroid-specific master regulator [71,72,77,78]. GATA-1 has been reported to regulate genes involved in many pathways, such as anti-apoptotic regulation, hemoglobin synthesis, cell signaling as well as the cell cycle [79].

**Figure 2:**
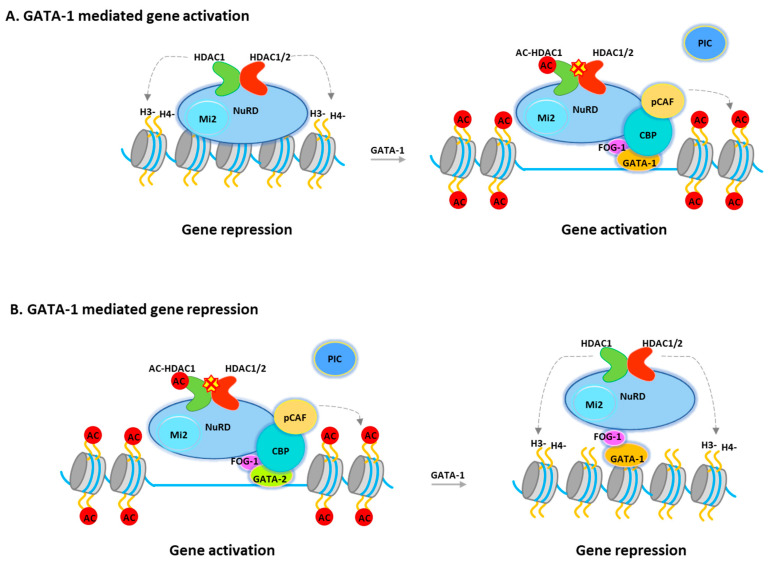
HDAC1 in NuRD complex mediates GATA-1 function. (**A**) A model for GATA-1 mediated gene activation. GATA-1 is recruited to the target sites and subsequently recruits NuRD complex and p300/CBP coactivators. These coactivators acetylate histones, GATA-1 and HDAC1, resulting in gene activation. (**B**) A model for GATA-1 mediated gene repression. GATA-1 displaces GATA-2 and recruits the NuRD complex with full deacetylase activity for repression. PIC, Pre Initiation Complex. PCAF, p300/CBP-associated factor.

The transcriptional activity of GATA-1 is regulated through interactions with a variety of transcription factors and cofactors. GATA-1 interacts with TAL1, KLF1, PU.1, and SP1 (Sp1 transcription factor) [80]. GATA-1 has also been reported to interact with chromatin remodeling/modification proteins, including the CBP/p300 histone acetyltransferases, and the SWI/SNF (SWItch/Sucrose Non-Fermentable) chromatin remodeling complexes [77,81]. Recent studies showed that GATA-1 is associated with NuRD or MeCP1 corepressor complexes through its interaction with FOG-1 [82,83,84] in either uninduced or induced terminally differentiated murine erytholeukemia (MEL) cells. It was suggested that these complexes play important roles in GATA-1 mediated repression of target genes such as GATA-2, c-Myc (Cellular myelocytomatosis oncogene), and c-kit (Mast/stem cell growth factor receptor Kit), which are all required for the proliferation of hematopoietic progenitors. However, the NuRD/MeCP1 complex is also recruited to GATA-1 sites, which mediate gene activation in induced cells, such as the β-globin gene, where GATA-1 binding also requires FOG-1 [48,82,83]. Hence, the question will be how the corepressor complex regulates GATA-1 mediated gene activation. In fact, GATA-1-associated HDAC1 is increasingly acetylated after differentiation [49]. Consistently, overexpression of an HDAC1 mutant, which mimics acetylated HDAC1, promotes GATA-1-mediated transcription and erythroid differentiation. Furthermore, during erythroid differentiation, acetylated HDAC1 recruitment is increased at GATA-1-activated genes while decreased at GATA-1-repressed genes. (Figure 2) Interestingly, deacetylase activity is not required for Mi-2 remodeling activity, suggesting that remodeling activity may be required for both gene activation and repression. Therefore, NuRD can function as a coactivator or a repressor and that acetylated HDAC1 converts the NuRD complex from a repressor to an activator during GATA-1-directed erythroid differentiation (Figure 2). Notably, the NuRD complex become MBD2 free during erythropoiesis. Therefore, the complex that acts as a transcriptional coactivator may have different configurations to facilitate gene activation [85]. 

GATA-1 can be acetylated at both N- and C-terminal zinc-fingers at conserved lysine residues. Acetylation of GATA-1 may or may not affect DNA binding depending on the GATA-1 binding sites [86,87]. Lysine to alanine mutations of acetylated sites affect chromatin binding, transcription activation and erythroid differentiation [88,89,90]. Acetylation can also target GATA-1 to ubiquitin dependent degradation [91]. It remains to be investigated whether acetylated GATA-1 can be dynamically deacetylated and whether NuRD or HDAC1 can deacetylate GATA-1. It has been reported that HDAC5 can interact with GATA-1 and repress GATA-1 mediated transcription [92], but there is no evidence that HDAC5 can deacetylate GATA-1. 

## 5. HDAC1 in Sin3 Complex Modulates TAL1 Function in Hematopoiesis

TAL1 is a member of the basic helix-loop-helix (bHLH) family of transcription factors and is required for the development of all hematopoietic cell lineages [93]. TAL1 forms heterodimer with the products of the ubiquitously expressed bHLH genes, *E2A* or *HEB*, and recognizes a hexanucleotide sequence CANNTG termed “E box” to act as regulators of transcription. Knockout of *Tal1* in mice leads to embryonic lethality at E8.5 due to complete loss of yolk sac hematopoietic cells [94,95]. Consistently, *Tal1* null embryonic stem cells are unable to generate both primitive and definitive erythroid cells in vitro and do not contribute to hematopoiesis in vivo in chimeric mice [93,96]. The data suggest a key role of TAL1 in hematopoiesis and subsequently, erythropoiesis. Interestingly, TAL1 associates with many transcription regulators, including GATA-1, LMO2, Ldb1, E2A, Bcl11a, NF-E2, KLF11 (Krüpple-like factor 11), BACH1 (BTB and CNC homolog 1), Myb (Myeloblastosis oncogene), and Myc [97]. All of these proteins have been implicated in the regulation of erythropoiesis. The TAL1/LMO2/GATA-1 containing protein complex specifically recognizes composite binding motifs consisting of a GATA site and an E-Box motif. This composite element is found in the regulatory regions of many erythroid-specific target genes including p4.2, KLF1, GATA-1, the locus control region (LCR) and the β-major globin promoter [98,99,100,101].

During hematopoiesis, TAL1 can function as a repressor or an activator of transcription depending on the DNA sequence and/or cellular contexts [102]. TAL1 recruits HDAC1/2-associated Sin3A corepressor complexes which mediate its transcription repressive activity in certain stages of erythroid differentiation [103]. The TAL1 associated deacetylase activity that appears to be important for proliferation of erythroid progenitors/precursors are markedly decreased during differentiation [103]. Inhibition of HDAC activities, such as treatment with TSA (Trichostatin A), a non-specific histone deacetylase inhibitor, synergizes with TAL1 to promote erythroid differentiation [103], suggesting that HDAC activity blocks erythroid progenitor cell differentiation via modulating TAL1 transcriptional activity. Consistent with the above findings, two groups recently undertook biochemical purification of TAL1-associated complexes from murine erythroleukemia (MEL) cells and showed that TAL1 is associated with the co-repressor ETO-2 (Eight twenty one protein, MTG8-related protein 2) [104,105]. Similar to its interaction with mSin3A, the interaction between TAL1 and ETO-2 decreased during erythroid differentiation of primary fetal liver cells [104]. Interestingly, ETO-2 also associates with HDACs and NCoR (Nuclear receptor corepressor) to repress target gene transcription [106,107,108]. 

## 6. HDAC1 Is Required for PU.1 Promoter Activation

PU.1 belongs to ETS (Erythroblast Transformation Specific) family of transcription factor that plays an essential role in hematopoietic stem cell self-renewal and in directing multipotent hematopoietic stem cells towards lineage commitment [79,109,110,111]. The expression of PU.1 fluctuates in various hematopoietic differentiation pathways. PU.1 protein level is high in human HSC cells [112,113,114,115] and remains high in common myeloid progenitors (CMPs), common lymphoid progenitors (CLPs), but changes to low as myeloid/erythroid precursor cells commit to the erythroid lineage [116,117,118,119]. Inappropriate expression of PU.1 in specific hematopoietic cells can result in leukemic transformation, such as T-cell lymphomas, acute myeloid leukemia and erythroleukemias [120,121,122]. Therefore, maintaining proper levels of PU.1 is critical for hematopoiesis. 

**Figure 3:**
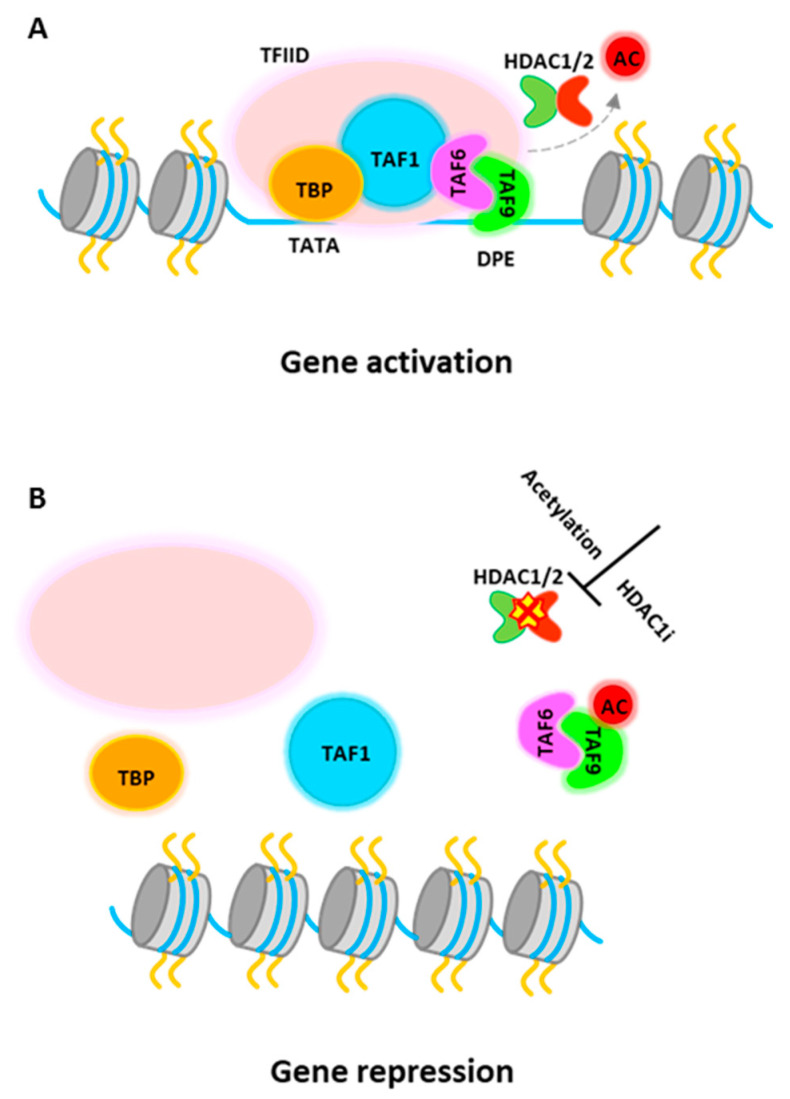
HDAC1 dependent dynamic transcription activation and repression at PU.1 promoter. (**A**) TAF9 can be acetylated by an acetyltransferase and subsequently deacetylated by HDAC1. Non-acetylated TAF9 binds to DPE site and associates with TFIID complex to promote PU.1 gene activation. (**B**) HDAC1 is acetylated or inactivated by HDACi, causing an inability to deacetylate TAF9. Acetylated TAF9 is displaced from DNA. Acetylated TAF9 also causes the disassembly of the TFIID complex from the promoter, resulting in gene repression.

PU.1 gene expression can be regulated epigenetically [5,123,124]. It is reported that inhibition of HDAC activity by HDACi treatment represses PU.1 gene expression [124], although it is generally considered that HDACs associate with corepressor complexes and repress gene transcription. Recent study further demonstrates that PU.1 directly requires histone deacetylase activity for gene activation [5]. HDAC1 is recruited to active PU.1 promoter in progenitor cells to promote PU.1 gene transcription. Detailed study shows that TATA-box binding protein associated factor 9 (TAF9), a member of the TFIID complex, is a target for HDAC1-mediated deacetylation and this process is required for PU.1 gene activation. The transcription factor IID (TFIID) is the first complex to be recruited to the core promoter element for preinitiation complex (PIC) assembly and that also serves to mediate signals between various activators and the basal transcription machinery [125,126,127]. TAF9 binds to a consensus DNA sequence, the downstream promoter element (DPE), which is found in both TATA-containing and TATA-less promoters, although more commonly in TATA-less promoters [128,129]. The study from Jian et al. shows that HDAC1 associates with TAF9 to maintain it in an unacetylated stage. The non-acetylated TAF9 is able to associate with the PU.1 promoter and promotes gene activation (Figure 3A). Upon induction of erythropoiesis, acetylated HDAC1 becomes accumulated at the PU.1 promoter and no longer be able to deacetylate TAF9. Acetylated TAF9 not only fails to bind to the promoter, but also results in the disassembly of the entire TFIID complex from the promoter, leading to transcriptional repression [5] (Figure 3B). Thus, this study demonstrate a key role of HDAC1 in PU.1 gene transcription and more importantly, the study shows that acetylation of HDAC1 during erythropoiesis has multiple roles, turning on transcription activators such as GATA-1 or other activators, and turning off counter regulators, such as PU.1.

In addition, HDAC1 can directly modulate PU.1 activity. It was found that the C-terminal domain of PU.1 formed a complex with mSin3A and HDAC1 in vivo, which mediates PU.1 induced transcriptional repression [130]. Further study shows that PU.1 directly associates with MeCP2 (Methyl CpG-binding protein 2) corepressor, which facilitates PU.1-mSin3A-HDAC complex formation. The complex disassociates from the region during the course of erythroid differentiation of MEL cells [131]. PU.1 itself can be acetylated and its repressor activity was reduced when the putative acetylation motifs in the Ets domain were mutated. The acetyl-mimicking mutant also insufficiently interacts with GATA-1 and mSin3A [132]. Therefore, HDAC1 plays important roles on the repressive function of PU.1 for erythroid differentiation blockage. 

## 7. KLF1 Regulates Erythroid Differentiation by Interacting with HDAC1 Containing Complexes

KLF1 (EKLF) is another transcription factor essential for erythropoiesis. KLF1 expression is restricted to hematopoietic compartment and expressed in both primitive and definitive erythroid populations [133]. Gene ablation studies showed that KLF1 null mice suffered profound β-thalassemia resulting in embryonic lethality [134,135].

KLF1 regulates erythropoiesis mainly through activation of gene transcription [133,136,137]. Genome wide analysis of KLF1 chromatin occupancy revealed that it binds to the proximity sites to GATA-1, suggesting co-occupation of these factors at the same promoter/enhance region [137].

Although KLF1 has been primarily characterized as a transcriptional activator, it can also exert transcriptional repression. p300/CBP (E1A binding protein p300/CREB-binding protein) can acetylate KLF1 at lysine 288. Acetylation leads to increased association of KLF1 with the chromatin remodeling SWI/SNF-related complex, resulting in an open chromatin domain and transcription of the adult β-globin promoter [138,139,140]. However, acetylation also promotes interaction with Sin3A and HDAC1 corepressors [141]. These studies suggest that dynamic coactivator-corepressor interaction through acetylation status of KLF1 modulates downstream transcriptional effects [133,139,140,142]. On the other hand, sumoylation of K74 facilitates KLF1 interaction with the Mi-2β of the NuRD repressor complex, which is important for suppression of megakaryocyte differentiation and direct MEP to erythroid lineage [143].

Interestingly, KLF1 also physically interact with TAF9, a component of TFIID complex. This interaction is required for a subset of KLF1 activated genes, including β-globin promoter, and is necessary for its activation. Since the interaction is not required at all KLF1 activation target promoters, it is conceivable that the requirement of TAF9 by KLF1 may depend on specific promoter sequences DPE, which is present at the β-globin promoter [144,145]. Further analysis is necessary to determine whether the DPE site is required for co-recruitment of TAF9 and KLF1. This is particularly interesting since TAF9 is modulated by HDAC1 in a DPE dependent manner [5]. 

## 8. Gfi-1b Repressed Erythroid and Megakaryocytic Differentiation through Association with CoREST Corepressor Complex

The family of Gfi-1 zinc finger oncoproteins consists of Gfi-1 and Gfi-1b. Studies from gene targeting and mutational screening in humans have revealed an essential role for Gfi-1 and Gfi-1b in hematopoiesis [146]. In mice, Gfi-1b is required for the development of two related blood lineages, erythroid and megakaryocytic. Disruption of Gfi-1b is embryonic lethal due to delayed maturation of primitive erythrocytes and failure to produce definitive enucleated erythrocytes [146,147]. Induced disruption of Gfi-1b at the adult stage was also lethal within 3 weeks after induction, with severely reduced hemoglobin levels and platelet counts. Genome-wide gene expression analyses revealed that Gfi-1b directly regulates a wide spectrum of megakaryocytic and erythroid genes, predominantly through gene repression [148]. Transcriptional repression by Gfi-1b requires the conserved SNAG (Snail/Gfi-1) domain. Gfi-1b associates with CoREST corepressor complex, which contains the corepressor CoREST, the histone demethylase LSD1, and HDAC1 and 2. Gfi-1b recruits these cofactors to the majority of target gene promoters in vivo. Inhibition of factors in CoREST complex perturbs differentiation of erythroid, megakaryocytic, and granulocytic cells as well as primary erythroid progenitors [149]. 

Gfi-1b p32 is an alternative spliced form that lacks the first two zinc finger domains of the protein. Gfi-1b p32 isoform binds to Gfi-1b target gene promoters and associates with the LSD1-CoREST repressor complex more efficiently than the major Gfi-1b p37 isoform. Selective knock down of Gfi-1b p32 compromises erythroid differentiation, further demonstrates that association of CoREST complex is essential for the repressive effect of Gfi-1b [150]. 

## 9. BCL11A Interacts with Multiple HDAC1/2 Containing Corepressor Complex for Fetal Globin Gene Suppression

BCL11A is a zinc finger transcription repressor. Genome-wide association studies (GWAS) have linked BCL11A to fetal globin level, which directly associates with the clinical severity of sickle cell disease and β-thalassemia [151,152]. Genetic study further shows that BCL11A is a repressor for fetal globin gene expression. Knockout of *Bcl11a* in mice prevents silencing of endogenous mouse fetal globin and transgenic human γ-globin [153]. Inducible deletion of *Bcl11a* in adult mice results in derepression of fetal globin, indicating that BCL11A actively repress fetal globin expression in the adult stage and that repression is reversible. Importantly, knock down of BCL11A in transgenic sickle cell disease mice stimulated therapeutic levels of fetal globin, suggesting the importance of BCL11A in hemoglobinopathy treatments [154].

BCL11A binds to the globin locus control region (LCR) and multiple other regions at the β-globin gene locus to regulate globin gene expression [155,156,157]. In adult erythroid cells, BCL11A interacts and co-operates with SOX6 (SRY-box transcription factor 6) in silencing γ-globin transcripts [157]. Liu et al. (2018) showed that BCL11A can directly bind to the γ-globin gene promoter to mediate γ-globin repression during hemoglobin switching [158]. BCL11A is found within multiprotein complexes consisting of erythroid transcription factors, transcriptional corepressors, and chromatin-modifying enzymes [156]. Affinity purification result shows that BCL11A associates with multiple corepressor complexes. These include members of the NuRD, LSD1/CoREST, NCoR/SMRT (Silencing mediator for retinoid and thyroid hormone receptors), SIN3, and SWI/SNF complexes [156]. Interestingly, these complexes bind to distinct sites at the globin locus and they all require HDAC1 and HDAC2 for repression. Knock down of HDAC1/2 significantly elevates fetal globin expression without significantly altering β-globin expression. 

## 10. EpoR/JAK2/Stat5 Signaling Requires HDAC1

Erythropoietin (EPO) is the primary hormone that regulates erythroid cell maturation. EPO binds to its cognate receptor EpoR and subsequently activates the Janus Kinase 2 (JAK2), which activates signaling pathways, including mitogen-activated protein kinase (MAPK), phosphatidylinositol 3-kinase (PI3 kinase), signal transducer and activator of transcription 5 (Stat5) [159,160]. EPO-stimulated STAT5 activation is essential and sufficient for erythropoiesis [161]. A genome wide mapping on EPO activated STAT5 recruitment on chromatin shows that STAT5 binds to various genes necessary for erythroid differentiation and maturation. In addition, STAT5 binds within the proximity of binding sites for other transcription factors, such as TAL1, GATA1, and KLF1, to direct erythroid differentiation [162]. How HDAC1 regulates STAT5 during erythroid differentiation is not fully studied. However, in murine T and B lymphocytes, treatment with HDACi represses transactivation of all cytokine induced STAT5 target genes. The deacetylase activity appear to be required to control proper assembly of the basal transcription machinery on STAT5 target genes and is independent of histone acetylation [163]. A detailed study on the role of HDAC1 was reported on STAT5 target gene Id1 (Inhibitor of DNA Binding 1). The study shows that both STAT5 and C/EBPβ (CCAAT-enhancer-binding protein beta) binding to Id1 promoter are required for Id1 transcription activation. HDAC1 physically interacts with STAT5 on Id1 promoter and subsequently deacetylate C/EBPβ. Deacetylation enable C/EBPβ to retain on chromatin and activate Id1 promoter activity [164]. It remains for future investigation whether deacetylation of C/EBPβ is a general mechanism for STAT5 mediated gene activation.

## 11. Use of Histone Deacetylase Inhibitor for Hemoglobinopathies

Sickle cell disease and β-thalassemia represent the most common hemoglobinopathies. The expression of normally silenced fetal globin in adult erythroid cells can ameliorate the pathophysiological consequences. Therefore, mechanisms of fetal globin reactivation have been intensively studied in the past few decades. 

In addition to factors such as BCL11A that mediate silencing of the γ-globin gene in adult stage erythroid cells, epigenetic factors, such as chromatin remodelers and histone modification enzymes have been intensively studied for their role in fetal globin gene expression [165,166]. The knock down of Mi-2β, the chromatin remodeler within NuRD corepressor complex, relieves γ-globin gene silencing in β-YAC (Yeast artificial chromosome) transgenic murine erythroid cells and in CD34(+) progenitor-derived human primary adult erythroid cells [167]. Depletion of MBD2, a component of the NuRD complex, increased γ to β mRNA ratios in adult erythroid cells [168]. γ-globin expression is also regulated by HDACs through chromatin modification, and several transcription factors that regulate HbF are associated with these HDAC proteins [166]. Indeed, HDAC1 and HDAC2 have been identified as potential new molecular targets for mediating fetal hemoglobin induction by chemical library screening [169]. The targeted inactivation of HDAC1 and HDAC2 is effective to enhance fetal globin expression in human adult erythroid cells without altering cell cycle [170]. Consistently, treatment with HDAC1/2 specific inhibitor Romidepsin [171] induces fetal globin expression. The combination of BCL11A inhibitor and Romidepsin further increased γ-globin gene expression [172]. MS-275 (Entinostat), a HDAC1/3 inhibitor, also increases γ-globin gene expression [173]. HDAC inhibitors can activate γ-globin expression via a p38 mitogen-activating protein kinase (MAPK)-dependent mechanism [174]. A recent report shows that selective HDAC inhibitors have been developed for the activation of γ-globin. The small molecule ACY-957 screened from a chemical library can selectively inhibit HDAC1 and HDAC2 and induces γ-globin transcripts and protein through activation of GATA-2 in primary erythroid progenitor cells [175]. 

Two clinical trials have been conducted with two pan-HDAC inhibitors for sickle cell disease therapy. One trial used Vorinostat and another trial used Panobinostat as a single drug for therapy (information received from clinicaltrials.gov). The Vorinostat treatment shows effective elevation of γ-globin level and increased γ to β globin ratios. However, the trial was terminated due to blood related adverse events [12,176], which further suggests that HDACi affects hematopoietic differentiation. More study is needed to evaluate the efficacy of the treatment and adverse effect of HDACi in the treatment. 

## 12. Closing Remark and Future Prospective

HDACs have been known to associate with many hematopoietic lineage specific transcription factors and mediate transcription regulation through these factors. GATA-1 and PU.1 are master regulators which functionally antagonize each other’s activity. While PU.1 directs myeloid differentiation, GATA-1 promotes erythroid differentiation (reviewed in [79]). HDAC1 plays an important role in counter regulating PU.1 and GATA-1 [5,49]. Deacetylase activity of HDAC1 represses GATA-1 activity but activates PU.1. During erythropoiesis, increased level of HDAC1 acetylation reduces deacetylase activity, which results in GATA-1 activation and PU.1 repression. Therefore, HDAC1 is an essential regulator of the master regulators for erythropoiesis.

The aberrant recruitment of HDAC1 is linked to various types of leukemia [177,178,179]. HDAC inhibitors (HDACi) have shown clinical and preclinical promise when used alone or in conjunction with other therapeutic drugs [180,181,182]. In addition, the inhibition of histone deacetylases results in the reactivation of fetal globin in adult erythroid cells [174,183,184,185,186,187]. Clinical trials have tested the effectiveness of HDACi for reactivation of fetal globin in patients suffering thalassemia and sickle cell diseases. However, blood related side effects led to termination of that study. Therefore, it is extremely important to understand how HDAC impacts normal hematopoiesis before HDACi is administered to patients.

## Abbreviations

BACH1	BTB and CNC homolog 1
BCL11A	B-cell lymphoma/leukemia 11A
BHC80	BRAF35-HDAC complex protein BHC80
bHLH	Basic helix-loop-helix
BRMS1	Breast cancer metastasis suppressor 1
C/EBP	CCAAT-enhancer-binding protein
CHD3/4	Chromodomain Helicase DNA binding protein 3/4
c-kit	Mast/stem cell growth factor receptor Kit
CLPs	Common lymphocyte progenitors
CMPs	Common myeloid progenitors
c-Myc	Cellular myelocytomatosis oncogene
CoREST	Corepressor of RE1 silencing transcription factor
CtBP	C-terminal binding protein
DPE	Downstream promoter element
E2A	Early 2A protein
EPO	Erythropoietin
ETO-2	Eight twenty one protein, MTG8-related protein 2
Ets	Erythroblast Transformation Specific
FOG-1	Friend of GATA Protein 1
GATA-1/2	GATA-binding factor 1/2
Gfi-1	Growth factor independent protein 1
GR	Glucocorticoid receptor
GWAS	Genome-wide association studies
HAT	Histone acetyltransferases
Hda1	Histone deacetylase HDA1
HDAC	Histone deacetylase
HDACi	HDAC inhibitors
HEB	Helix-loop-helix, E-box-binding protein
HSC	Hematopoietic stem cells
Id1	Inhibitor of DNA Binding 1
IL-4	Interleukin 4
ING1	Inhibitor of Growth Family Member 1
JAK2	Janus Kinase 2
K	Lysine
KLF1	Krüpple-like factor 1
KLF11	Krüpple-like factor 11
LCR	Locus control region
Ldb1	LIM-domain-binding protein 1
LMO2	LIM Domain Only 2
LSD1	Lysine-specific demethylase 1
Mad	Max dimerization protein
MAPK	Mitogen-activated protein kinase
Max	Myc-associated factor X
MBD2	Methyl CpG-binding domain 2
MeCP1/2	Methyl CpG-binding protein 1/2
MEL	Murine erytholeukemia
MEP	Megakaryocyte/Erythroid progenitor
Mi-2	ATP-dependent helicase Mi-2
MKP-3	Mitogen-activated protein kinase phosphatase-3
MPPs	Multipotent progenitors
MS-275	Entinostat, HDAC1 and 3 inhibitor
MTA	Metastasis-associated protein
MxCre	Mx dynamin-like GTPase/Cyclic recombinase
Myb	Myeloblastosis oncogene
NAD	Nicotinamide adenine dinucleotide
NCoR	Nuclear receptor corepressor
NF-E2	Nuclear factor, erythroid-derived 2
NuRD	Nucleosome remodeling and deacetylase
p300/CBP	E1A binding protein p300/CREB-binding protein
PAH	Paired amphipathic helix
pI/pC	Polyinosinic/polycytidylic acid
PI3 kinase	Phosphatidylinositol 3-kinase
PIC	Pre Initiation Complex
PU.1	Putative oncogene Spi-1
RbAp46/48	Retinoblastoma-associated proteins 46/48
RBP1	Retinoblastoma-binding protein 1
Rpd3	Reduced potassium dependency 3
SAP18	Sin3A Associated Protein 18
SAP180	Sin3A Associated Protein 180
SAP30	Sin3A Associated Protein 30
SCL	Stem cell leukemia
SDS3	Suppressor of defective silencing 3
Sin3	Switch Independent 3
Sin3A	SIN3 Transcription Regulator Family Member A
Sin3B	SIN3 Transcription Regulator Family Member B
Sir2	Silent information regulator 2
SMRT	Silencing mediator for retinoid and thyroid hormone receptors
SNAG	Snail/Gfi-1
SOX6	SRY-box transcription factor 6
SP1	Sp1 transcription factor
STAT5	Signal transducer and activator of transcription 5
SWI/SNF	SWItch/Sucrose Non-Fermentable
TAF9	TATA-Box Binding Protein Associated Factor 9
TAL1	TAL BHLH Transcription Factor 1
TFIID	Transcription factor IID
Th2	Type 2 helper T cells
TSA	Trichostatin A
YAC	Yeast artificial chromosome
	
